# Optimal media reporting intensity on mitigating spread of an emerging infectious disease

**DOI:** 10.1371/journal.pone.0213898

**Published:** 2019-03-21

**Authors:** Weike Zhou, Yanni Xiao, Jane Marie Heffernan

**Affiliations:** 1 Department of Applied Mathematics, School of Mathematics and Statistics, Xi’an Jiaotong University, Xi’an 710049, PR China; 2 Department of Mathematics & Statistics, York University, Toronto, Canada; Imperial College London, UNITED KINGDOM

## Abstract

Mass media reports can induce individual behaviour change during a disease outbreak, which has been found to be useful as it reduces the force of infection. We propose a compartmental model by including a new compartment of the intensity of the media reports, which extends existing models by considering a novel media function, which is dependent both on the number of infected individuals and on the intensity of mass media. The existence and stability of the equilibria are analyzed and an optimal control problem of minimizing the total number of cases and total cost is considered, using reduction or enhancement in the media reporting rate as the control. With the help of Pontryagin’s Maximum Principle, we obtain the optimal media reporting intensity. Through parameterization of the model with the 2009 A/H1N1 influenza outbreak data in the 8th Hospital of Xi’an in Shaanxi Province of China, we obtain the basic reproduction number for the formulated model with two particular media functions. The optimal media reporting intensity obtained here indicates that during the early stage of an epidemic we should quickly enhance media reporting intensity, and keep it at a maximum level until it can finally weaken when epidemic cases have decreased significantly. Numerical simulations show that media impact reduces the number of cases during an epidemic, but that the number of cases is further mitigated under the optimal reporting intensity. Sensitivity analysis implies that the outbreak severity is more sensitive to the weight *α*_1_ (weight of media effect sensitive to infected individuals) than weight *α*_2_ (weight of media effect sensitive to media items).

## Introduction

Emerging and reemerging infectious diseases including the 2003 severe acute respiratory syndrome (SARS) and the 2009 A/H1N1 influenza epidemic have become a major cause of mortality and morbidity in emergency situations. News reports have the potential to modify a community’s knowledge of emerging infectious diseases, and affect people’s attitudes and behaviours during infectious disease outbreaks [1, 2]. People informed by media reports can take precautions ranging from washing hands, wearing protective masks to avoiding social contact with infected individuals, to reduce their susceptibility. Informed infective individuals will also take measures to protect themselves from being exposed to others to reduce infectivity. It has been shown that behaviour change during infectious disease outbreaks can curb the effects of infectious diseases in populations [3].

In recent years, a growing number of studies have focused on understanding and quantifying the impact of such behaviour influencing factors on the spread of infectious diseases [4–18]. A number of studies have employed mathematical models to assess the impact of media reports on emerging infectious disease prevention and control [4, 5, 19–30]. Recently, Greenhalgh et al. [19] presented a brief and nice commentary on the literature related to awareness and their effects on the dynamics of diseases. In summary, it has been found that there are three main methods being used to incorporate behaviour change in mathematical models due to awareness of disease. In the first method, the incidence rate of the disease is reduced by some factors that depend on the numbers of infected individuals, hospitalized individuals or exposed individuals, due to education about preventative knowledge of the disease through media coverage. The common choice of the reduction factors is a saturated [4, 6, 31, 32] or exponential [20, 24, 27] growth function. For example, Liu et al. [27] incorporated an exponential decreasing factor *β*_0_ = *βe*^−*a*_1_*E*−*a*_2_*I*−*a*_3_*H*^ into the transmission coefficient (with exposed (E), infectious (I), hospitalized (H)) to illustrate the possible mechanism for multiple outbreaks of *SARS* due to the psychological impact. Cui et al. [32] used the general nonlinear incidence function *μ*_1_ − *μ*_2_ *f*(*I*) to represent the media and education impact on the spread of the infectious disease. In [5], the authors focused on simple endemic models by modelling the contact rate as a function of the available information on the present and the past disease prevalence.

In the second method, a separate compartment that effectively represents the level of awareness in the population is introduced, and individuals in the population can move from the unaware to aware compartments [7, 15, 16, 19, 21–23, 28]. For example, in [19], the authors proposed a mathematical model by inducing behavioural changes in the population through delineation of the susceptible class into unaware susceptible and aware susceptible subpopulations. [16] and [29] explicitly introduced distinct compartments for unaware and aware individuals in each of the disease states, and transitions between respective unaware and aware compartments took place at constant rates.

In the third method, a compartment representing the awareness program is incorporated [8, 18, 21, 25]. Yan and Tang et al. [25] described the effects of media reports on population infection by modifying the transmission rate *β* following an exponential function *βe*^−*pM*^ with *M* representing the level of media reports. Further, in [8], the media reporting is introduced as a separate compartment in a mathematical model and the susceptible population is divided into three awareness levels, each with a different infection rate. In [18], the authors considered the interaction of disease outbreak and media impact by formulating a susceptible-infected-hospitalized-recovered framework of population. By extension, susceptible and infected populations are subdivided into aware and unaware since individuals modified their behaviors to reduce their transmissibility and infectivity, and the dynamics of media reports was incorporated by considering how media was influenced by the numbers of infected and hospitalized individuals.

The majority of the mathematical modelling studies described above have incorporated media impact either in the disease transmission term or by dividing the susceptible population into subgroups with various awareness levels. However, the relationship between mass media and disease spread can be more complex than these models portray. On one hand, media reporting influences the public awareness of the disease and affects the effectiveness of prevention measures. On the other hand, the severity of the disease has an impact on the degree of mass media reporting. We’ve known that, in the first and third methods, media impact is modelled through the inclusion of a “media function”, which is proportional to the number of infected individuals and/or the level of media reports, to reduce the incidence rate through increased protective behaviour. However, it remains unclear as to whether awareness of the number of infections, or the awareness of media reports best modify individual behaviour during an infectious disease outbreak. This falls within the scope of this study.

Herewithin, we establish a mathematical model incorporating media reports as a separate compartment by considering how media is influenced by disease statistics (number of newly observed individuals). Disease progression is characterized by an SEIR model of which the transmission rate is modified by a media function affected by the media reports and also the number of infected individuals. The model can be recognized as the combination of the first method and the third method of modelling. In our model, we formulate the novel media function *f*(*I*, *M*, *α*_1_, *α*_2_) with *α*_*i*_ (*i* = 1, 2) denoting the weight of infected individuals and media reports, and examine their effects on disease spread. Further, we investigate an optimal control problem in order to seek the optimal reporting intensity of information to minimize the number of infected individuals (and costs). We parameterize the proposed model on the basis of the 2009 A/H1N1 data in Shaanxi province of China, and estimate the basic reproduction number and other unknown parameters. A sensitivity analysis is conducted to identify model parameters that most affect the peak magnitude of the epidemic, as well as the total number of infections over the entire epidemic.

## Methods

### Model

We are interested in studying the effects of *I* and *M* on the outcomes of an infectious disease outbreak/epidemic. We therefore consider a mass media compartment *M* and a media effect function that depends on both the number of infected *I* and media reports *M*. Consider an SEIR model that incorporates a compartment of media programs *M*, in which the media impact on the human behaviour is reflected in the contact rate.

$$\{\begin{array}{c} \frac{dS}{dt}=\Lambda -f\left(I,M,\alpha_{1},\alpha_{2}\right)\beta SI-\mu S,\\ \frac{dE}{dt}=f\left(I,M,\alpha_{1},\alpha_{2}\right)\beta SI-\sigma E-\mu E,\\ \frac{dI}{dt}=\sigma E-\gamma I-\mu I,\\ \frac{dR}{dt}=\gamma I-\mu R,\\ \frac{dM}{dt}=\rho \sigma E-\delta M. \end{array}$$ (1)

where *S*(*t*), *E*(*t*), *I*(*t*), *R*(*t*) represent the susceptible, exposed, infective, and recovered populations, respectively, *M*(*t*) represents the number of news items. Here, Λ is the birth rate, *μ* is the natural death rate, *σ* is the progression rate from the exposed to infective classes, and *γ* is the recovery rate. The propagation of information depends on the number of newly observed individuals (*σE*), and *ρ* represents the reporting rate of the newly observed individuals. It is assumed that *δ* represents the spontaneous disappearance rate of media. The baseline transmission rate without media effect is represented by *β* and *f*(*M*, *I*, *α*_1_, *α*_2_) is used to modify the transmission rate, which is induced by the media effect. Finally, *α*_1_, *α*_2_ are the weights of media effect sensitive to infectives and media items, respectively. All the parameters are non-negative.

It’s obvious that the media impact on the behavior of humans increases as the number of infected individuals increases or the intensity of media reports increases. Thus the term of media impact factor reflecting the behavior change *f*(*I*, *M*, *α*_1_, *α*_2_) is a decreasing function with respect to the number of infected individuals *I* and the media intensity *M*, it should satisfy the following assumptions:

$$\begin{array}{c} \begin{array}{c} \frac{\partial f(I,M,\alpha_{1},\alpha_{2})}{\partial I}\leq 0\;\text{for}\;\text{all}\;\;I>0,\;\;\frac{\partial f(I,M,\alpha_{1},\alpha_{2})}{\partial M}\leq 0\;\text{for}\;\text{all}\;\;M>0,\\ f\left(0,0,\alpha_{1},\alpha_{2}\right)=1,\;\;f\left(I,M,\alpha_{1},\alpha_{2}\right)\to 0\;\text{as}\;\;I\to \infty \;\;\text{or}\;\;M\to \infty . \end{array} \end{array}$$ (2)

Here, we choose two different media functions,

$$\begin{array}{c} f_{1}\left(I,M,\alpha_{1},\alpha_{2}\right)=e^{-\alpha_{1}I-\alpha_{2}M}, \end{array}$$

and

$$\begin{array}{c} f_{2}\left(I,M,\alpha_{1},\alpha_{2}\right)=\frac{1}{1+\alpha_{1}I+\alpha_{2}M}. \end{array}$$

### Optimal control

In system (1), the reporting rate is proportional to the newly reported number of infected individuals. It is natural to ask whether we should enhance the reporting rate to reduce the total infected number and minimize the cost of media reporting. Thus our main purpose is to minimize the total number of infective individuals as well as the cost required to reduce or enhance the media reporting intensity.

Consider the optimal control problem to minimize the objective functional

$$\begin{array}{c} \begin{array}{c} J\left(u\right)=\int_{t_{0}}^{T}\left[AI\left(t\right)+\frac{B}{2}u^{2}\left(t\right)\right]dt \end{array} \end{array}$$ (3)

subject to

$$\{\begin{array}{c} \frac{dS}{dt}=\Lambda -\beta f\left(I,M\right)SI-\mu S,\;\;S\left(t_{0}\right)=S_{0}>0,\\ \frac{dE}{dt}=\beta f\left(I,M\right)SI-\sigma E-\mu E,\;\;E\left(t_{0}\right)=E_{0}\geq 0,\\ \frac{dI}{dt}=\sigma E-\gamma I-\mu I,\;\;I\left(t_{0}\right)=I_{0}\geq 0,\\ \frac{dR}{dt}=\gamma I-\mu R,\;\;R\left(t_{0}\right)=R_{0}\geq 0,\\ \frac{dM}{dt}=u\left(t\right)\rho \sigma E-\delta M,\;\;M\left(t_{0}\right)=M_{0}\geq 0. \end{array}$$ (4)

where the coefficients *A* and *B*/2 are positive. Here we assume that *A* = 1, and that *B*/2 is the weight associated with the control *u*(*t*). Note that *u*(*t*) is a Lebesgue measurable function on a finite interval [*t*_0_, *t*_*end*_], where 0 ≤ *u*(*t*) ≤ *u*_*max*_, *u*_*max*_ > 1, and 0 ≤ *u*(*t*) < 1 represents reduction in reporting intensity, whereas 1 < *u*(*t*) ≤ *u*_*max*_ represents enhancement in reporting intensity.

### Parameter values

We use data from the 8th hospital of Xi’an in Shaanxi province to study the effects of media reports. The data are fully available in S2 File. The data include information on the daily number of hospital notifications in the 8th hospital from September 3 to 30, 2009. Parameter values for Eq (1) are informed by the literature and are further estimated through model fits to the hospital notification data, using the Least Square Method.

To ensure that our model estimates of the basic reproduction number, *R*_0_, are from the exponential growth phase of infection, we assume data from September 3-21 [33]. Also, so that we can compare the effects of *I* and *M* on the optimal control of media reporting, we assume a mass media compartment and a media function that depends both on *I* and *M*. Table 1 lists the best-fit parameters determined for model (1), without media impact *f*(*I*, *M*, *α*_1_, *α*_2_) = *f*_0_(*I*, *M*) = 1 and with two different media functions *f*_1_ = *e*^−*α*_1_*I*−*α*_2_*M*^ and $f_{2}=\frac{1}{1+\alpha_{1}I+\alpha_{2}M}$, respectively.

**Table 1:** Values of initial populations and parameters in the model (1).

Variables	Description	Initial value			Resource
		*f*_0_	*f*_1_	*f*_2_	
*S*(*t*)	Susceptible population	28410	28410	28410	LS
*E*(*t*)	Exposed population	59	59	61	LS
*I*(*t*)	Infected population	4	4	4	data
*R*(*t*)	Recovered population	0	0	0	[25]
*M*(*t*)	Media items	8	8	8	[25]
Parameters	Description	Value			Resource
		*f*_0_	*f*_1_	*f*_2_	
Λ	Birth rate of the population (per day)	0	0	0	-
*μ*	Natural death rate of the population (per day)	0	0	0	-
*β*	Contact transmission rate (per person per day)	0.0000154	0.0000158	0.0000158	LS
*α*_1_	Weight of media effect sensitive to infected individuals	0	0.00015	0.00015	LS
*α*_2_	Weight of media effect sensitive to media items	0	0.0138	0.0122	LS
*σ*	Progression rate from exposed to infected (per day)	1/2.8	1/2.8	1/2.8	[34]
*γ*	Recovery rate of infected population (per day)	1/4.16	1/4.16	1/4.16	[34]
*ρ*	Media reporting rate (per day)	-	0.01	0.01	LS
*δ*	Media waning rate (per day)	-	0.4940	0.2535	LS

Note that, for completeness, we consider the extended dataset from September 3-30 in a sensitivity analysis. Also, we have provided model fits and parameter values when the mass media compartment *M* is not included in the model in S1 File Appendix C. However, as we are interested in comparing the effects of *I* and *M* in the current study, we consider the full model with the *M* compartment in the optimal control and sensitivity analysis.

## Results

### Equilibrium

The basic reproduction number for system (1) can be calculated as

$$\begin{array}{c} R_{0}=\frac{\beta \sigma \Lambda }{\mu (\gamma +\mu )(\sigma +\mu )} \end{array}$$ (5)

easily using the next generation method [35] or the survival function method (see [36] for a review of this method and other methods that are commonly used). Note that the basic reproduction number is independent of the mass media compartment. Also note that it is not affected by the media function *f*(*I*, *M*). Therefore, it is the same as it would be in a model without media impact, which means that media coverage does not play a role in affecting this epidemic threshold. This has also been observed in previous studies [4, 6, 11].

Omitting the equation for *R*, the system (1) can be rewritten as a four dimensional model. Here, the disease free equilibrium is $E_{0}\left(\frac{\Lambda }{\mu },0,0,0\right)$, and the endemic equilibrium $E^{*}\left(S^{*},E^{*},I^{*},M^{*}\right)$ should satisfy

$$\{\begin{array}{c} \Lambda -f\left(I^{*},M^{*}\right)\beta S^{*}I^{*}-\mu S^{*}=0,\\ f\left(I^{*},M^{*}\right)\beta S^{*}I^{*}-\sigma E^{*}-\mu E^{*}=0,\\ \sigma E^{*}-\gamma I^{*}-\mu I^{*}=0,\\ \rho \sigma E^{*}-\delta M^{*}=0, \end{array}$$ (6)

Simplifying gives

$$\begin{array}{c} \begin{array}{c} S^{*}=\frac{\Lambda }{\mu }-\frac{\delta (\sigma +\mu )}{\rho \sigma \mu }M^{*},\;\;E^{*}=\frac{\delta }{\rho \sigma }M^{*},\;\;I^{*}=\frac{\delta }{\rho (\gamma +\mu )}M^{*}, \end{array} \end{array}$$ (7)

and

$$\begin{array}{c} \begin{array}{c} f\left(I^{*},M^{*}\right)\left(\Lambda -\frac{\delta (\sigma +\mu )}{\rho \sigma }M^{*}\right)=\frac{\mu (\sigma +\mu )(\gamma +\mu )}{\beta \sigma }. \end{array} \end{array}$$ (8)

Since I* can be expressed by M*, we can rewrite *f*(I*, M*) as $h\left(M^{*}\right)=f\left(\frac{\delta }{\rho (\gamma +\mu )}M^{*},M^{*}\right)$. Denote $h\left(M\right)=f\left(\frac{\delta }{\rho (\gamma +\mu )}M,M\right)$, then *h*(*M*) is a decreasing function with respect to *M*. Let

$$\begin{array}{c} \begin{array}{c} g\left(M\right)=h\left(M\right)\left(\Lambda -\frac{\delta (\sigma +\mu )}{\rho \sigma }M\right)-\frac{\mu (\sigma +\mu )(\gamma +\mu )}{\beta \sigma }. \end{array} \end{array}$$ (9)

Then we have $g\left(0\right)=\Lambda -\frac{\mu (\sigma +\mu )(\gamma +\mu )}{\beta \sigma }>0$ when $R_{0}>1$, $g\left(\frac{\rho \sigma \Lambda }{\delta (\sigma +\mu )}\right)=-\frac{\mu (\sigma +\mu )(\gamma +\mu )}{\beta \sigma }<0$, and $g^{\prime }\left(M\right)=h^{\prime }\left(M\right)\left(\Lambda -\frac{\delta (\sigma +\mu )}{\rho \sigma }M\right)-\frac{\delta (\sigma +\mu )}{\rho \sigma }h\left(M\right)<0$ holds true for any *M* satisfying $\Lambda -\frac{\delta (\sigma +\mu )}{\rho \sigma }M>0$. Thus there must exist one and only one positive root $M^{*}<\frac{\rho \sigma \Lambda }{\delta (\sigma +\mu )}$ that satisfies *g*(M*) = 0 if $R_{0}>1$. Particularly, if we choose

$$\begin{array}{c} f_{1}\left(I,M\right)=e^{-\alpha_{1}I-\alpha_{2}M}, \end{array}$$

and

$$\begin{array}{c} f_{2}\left(I,M\right)=\frac{1}{1+\alpha_{1}I+\alpha_{2}M}, \end{array}$$

we have

$$\begin{array}{c} M^{*}=\frac{\rho \sigma \Lambda }{\delta (\sigma +\mu )}-\frac{\rho (\gamma +\mu )}{\alpha_{1}\delta +\alpha_{2}\rho \left(\gamma +\mu \right)}LambertW\left(\frac{\mu (\alpha_{1}\delta +\alpha_{2}\rho \left(\gamma +\mu \right))}{\beta \delta }e^{\frac{(\alpha_{1}\delta +\alpha_{2}\rho \left(\gamma +\mu \right))\sigma \Lambda }{\delta (\sigma +\mu )(\gamma +\mu )}}\right), \end{array}$$

and

$$\begin{array}{c} M^{*}=\frac{\rho \mu \left(\gamma +\mu \right)\left(R_{0}-1\right)}{\mu (\alpha_{1}\delta +\alpha_{2}\rho \left(\gamma +\mu \right))+\beta \delta }, \end{array}$$

respectively. (Please find detailed definition of Lambert W function in paper [10, 37]).

Note that the disease free equilibrium $E_{0}\left(\frac{\Lambda }{\mu },0,0,0\right)$ is globally asymptotically stable if $R_{0}\leq 1$, and unstable if $R_{0}>1$. Meanwhile, the unique endemic equilibrium $E^{*}\left(S^{*},E^{*},I^{*},M^{*}\right)$ exists if and only if $R_{0}>1$ and it is locally asymptotically stable if it is feasible. For more information about the stability of the disease free equilibrium and the endemic equilibrium, see S1 File Appendix A.

### Existence of optimal control

Denote the control set $U=\{u\left(t\right):0\leq u\left(t\right)\leq u_{max},t_{0}\leq t\leq t_{end},u\left(t\right)\;\text{is}\;\text{Lebesgue}\;\text{measurable}\}$. The existence of optimal control can be shown using the results from Theorem 4.1 in [38]. We can easily verify the following properties:

1. The set of control and corresponding state variable is non-empty, which can be shown by the boundedness of solutions of system (4) using the results from Theorem 9.2 in [39].
2. The control set U is closed and convex by definition.
3. The right-hand side of the state system is bounded above by a linear function in the state and control, since the solutions are bounded, which determines the compactness needed for the existence of the optimal control.
4. The integrand of the objective functional is convex on the control *u*(*t*), and there exists *q*_1_ > 0, *q*_2_ > 1 such that $AI\left(t\right)+B/2u{\left(t\right)}^{2}\geq q_{1}{\left|u\left(t\right)\right|}^{q_{2}}-q_{3}$, where we can choose *q*_1_ = *B*/2 and *q*_2_ = 2.

Then we have that, for the control problem (3) and (4), there exists an optimal control $u^{*}\in U$ such that min *J*(*u*) = *J*(u*) on the interval [*t*_0_, *t*_*end*_].

**Theorem 1** There exists an optimal control u* that minimizes J(*u*) *over* U. *Moreover, there exists adjoint functions*

$$\{\begin{array}{c} \lambda_{S}^{\prime }\left(t\right)=\beta f\left(I,M\right)I\left(\lambda_{S}-\lambda_{E}\right)+\mu \lambda_{E},\\ \lambda_{E}^{\prime }\left(t\right)=\sigma \left(\lambda_{E}-\lambda_{I}-\rho u\left(t\right)\lambda_{M}\right)+\mu \lambda_{E},\\ \lambda_{I}^{\prime }\left(t\right)=-A+\beta f\left(I,M\right)S\left(\lambda_{S}-\lambda_{E}\right)+\beta SI\frac{\partial f(I,M)}{\partial I}\left(\lambda_{S}-\lambda_{E}\right)+\gamma \left(\lambda_{I}-\lambda_{R}\right)+\mu \lambda_{I},\\ \lambda_{R}^{\prime }\left(t\right)=\mu \lambda_{R},\\ \lambda_{M}^{\prime }\left(t\right)=\beta SI\frac{\partial f(I,M)}{\partial M}\left(\lambda_{S}-\lambda_{E}\right)+\delta \lambda_{M}, \end{array}$$ (10)

*with the transversality conditions*

$$\begin{array}{c} \lambda_{S}\left(t_{end}\right)=0,\lambda_{E}\left(t_{end}\right)=0,\lambda_{I}\left(t_{end}\right)=0,\lambda_{R}\left(t_{end}\right)=0,\lambda_{M}\left(t_{end}\right)=0. \end{array}$$ (11)

The optimal control u* is given by

$$\begin{array}{c} u^{*}\left(t\right)=\max\left\{\min\left\{\frac{-\lambda_{M}\rho \sigma E}{B},u_{max}\right\},0\right\}. \end{array}$$ (12)

For detailed derivation of the optimal control for the control problem (3) and (4), see S1 File Appendix B.

### Numerical simulation

**Fig 1:**
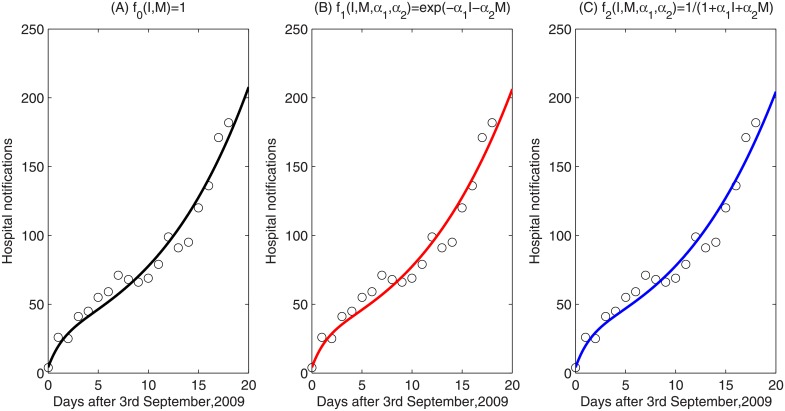
Data fitting for the daily number of hospital notifications from September 3rd to 21st, 2009 in the 8th hospital of Xi’an. Circles represent the hospital notifications in the 8th hospital of Xi’an, black curve, red curve and blue curve represent the estimating solutions for the number of infected individuals in system (1) without media impact (i.e. *f*_0_ = 1), and by using media function *f*_1_ = *e*^−*α*_1_*I*−*α*_2_*M*^, $f_{2}=\frac{1}{1+\alpha_{1}I+\alpha_{2}M}$, respectively.

During the 2009 A/H1N1 influenza pandemic, media coverage was used to spread precaution information about the disease, which influenced human behaviours [11, 25]. Using data extracted from the initial laboratory-confirmed cases of 2009 A/H1N1 that were admitted to the 8th hospital of Xi’an for the period 3rd September to 21st September, and the Least Square Method, we estimated the parameters shown in Table 1 and Fig 1, with R-square value being 0.9588, 0.9577, 0.9583, using three different media functions *f*_0_, *f*_1_, *f*_2_. Note that the goodness of fit is significant, but similar for each case. This is not unexpected as the data spans 19 days only. Also, however, as shown in our discussion of *R*_0_, during the early stages of an epidemic there is little effect due to mass media reports. Therefore it is reasonable that the model fits and parameter values are similar comparing the model with (*f*_1_, or *f*_2_) and without (*f*_0_) media.

Using the Akaike Information Criterion (*AIC*) for Least-Squares case, $AIC=n\log(\frac{RSS}{n})+2k$, where *n* is the sample size, *k* is the number of parameters, and *RSS* denotes residual sum of squares of fitted model, we obtain an *AIC* of 90.8918 for the model without media impact (i.e. the model with *f*_0_), 99.3676 for the model with *f*_1_, and 99.0981 for the model with *f*_2_. We note that the model without media impact has the lowest *AIC* and models that do not consider the mass media compartment *M* also fit the data well (see S1 File Appendix C). However, as we are interested in understanding the effects of mass media reports *M* and known infectives *I* to an individual in the population, we continue our study considering the model with the *M* compartment, and media functions *f*_1_ and *f*_2_. Considering the R-square and *AIC*, we conclude that model (1) with *f*_2_ fits the observed data better than the model with media function *f*_1_.

**Fig 2:**
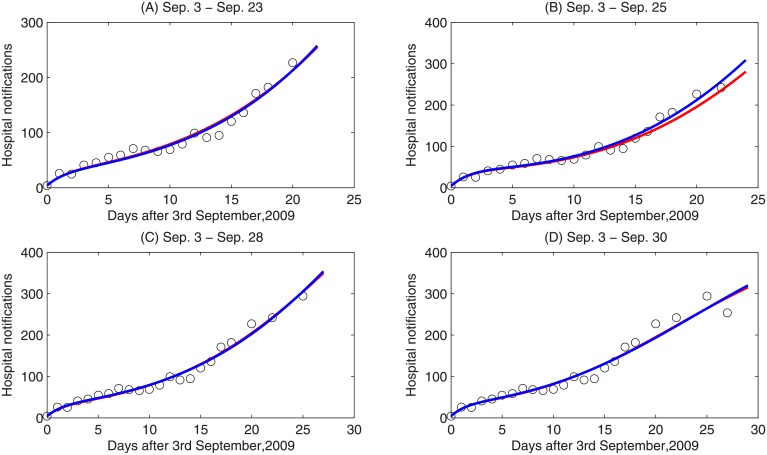
Data fitting for four time intervals. (A) September 3-23; (B) September3-25; (C) September 3-28; (D) September 3-30, 2009. Circles represent the hospital notifications in the 8th hospital of Xi’an, red and blue curves are the fitting curves for the model with *f*_1_ and *f*_2_, respectively.

To show the sensitivity of the basic reproduction number *R*_0_ with respect to the time interval considered, we also estimated key epidemic parameters and initial conditions considering the time periods between September 3rd and 23rd, 25th, 28th, and 30th, respectively. Results are presented in Table 2 and shown in Fig 2. When different periods are considered, the reproduction number varies from 1.8715 to 2.0463. The results indicate that, for each period we consider, the model with both *f*_1_ and *f*_2_ can fit the observed data well. Note that we have chosen to consider the model fits using data from the 3rd to 21st of September in the analysis below. This is done to ensure that we estimate *R*_0_ during the exponential growth phase of the outbreak [33].

**Table 2:** Parameter estimates based on data from the 8th hospital of Xi’an after 3rd September.

Parameter	Sep. 3-21		Sep. 3-23		Sep. 3-25		Sep. 3-28		Sep. 3-30	
	*f*_1_	*f*_2_	*f*_1_	*f*_2_	*f*_1_	*f*_2_	*f*_1_	*f*_2_	*f*_1_	*f*_2_
*S*(0)	28410	28410	28970	29000	29512	29510	28483	28558	29352	29351
*E*(0)	59	61	58	57	77	79	60	62	66	66
*β*	1.58e-5	1.58e-5	1.583e-5	1.58e-5	1.6622e-5	1.6571e-5	1.6401e-5	1.5947e-5	1.68e-5	1.672e-5
*α*_1_	1.5e-4	1.5e-4	1.5e-4	1.5e-4	1.9691e-4	1.7433e-4	4.9951e-4	3.6903e-4	0.0011	0.0013
*α*_2_	0.0138	0.0122	0.01	0.01	0.1124	0.1782	0.0166	0.0146	0.0598	0.0563
*ρ*	0.01	0.01	0.0352	0.01	0.0032	0.0024	0.01	0.01	0.01	0.01
*δ*	0.4940	0.2535	0.5	0.2940	0.2616	0.3086	0.2914	0.2577	0.4469	0.4481
*R*_0_	1.8715	1.8716	1.9118	1.9101	2.0463	2.0401	1.9477	1.8989	2.0563	2.0589
R-square	0.9577	0.9583	0.9663	0.9687	0.9796	0.9787	0.9803	0.9797	0.9602	0.9583
*AIC*	99.3676	99.0981	106.6231	105.1710	107.4610	107.3906	117.3241	117.9277	140.9177	141.999

Using data from September 3-21, the basic reproduction number is estimated as *R*_0_ = 1.8248 without media impact (*f*(*I*, *M*, *α*_1_, *α*_2_) = *f*_0_ = 1), which is lower than the estimates for *R*_0_ = 1.8715 for media function *f*(*I*, *M*, *α*_1_, *α*_2_) = *f*_1_ and 1.8716 for media function *f*(*I*, *M*, *α*_1_, *α*_2_) = *f*_2_. These three values of *R*_0_ are all in agreement with the result in [25], in which the mean value of the basic reproduction number was estimated as 1.794 with 95% confidence interval [1.3858, 1.9091]. Again, we note that the similar *R*_0_ values reflect the fact that there is little impact of media reports in the early days of the epidemic.

**Fig 3:**
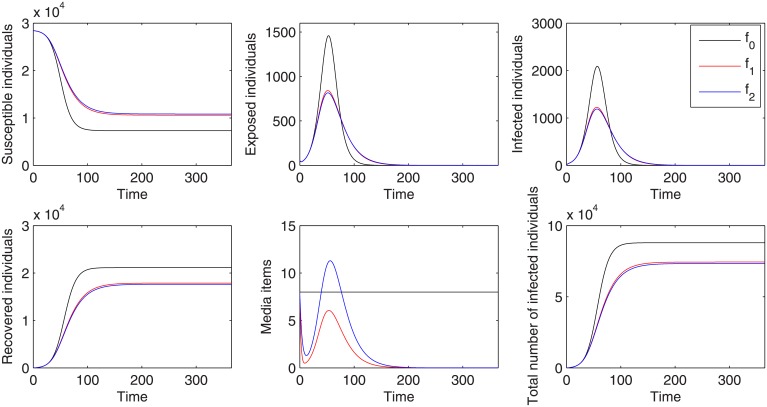
Comparison of solutions for system (1) with and without media effect. Black, red, blue curves represent the result of without media effect (*f*_0_ = 1), with media function *f*_1_ = *e*^−*α*_1_*I*−*α*_2_*M*^, and with media function $f_{2}=\frac{1}{1+\alpha_{1}I+\alpha_{2}M}$, respectively.

**Fig 4:**
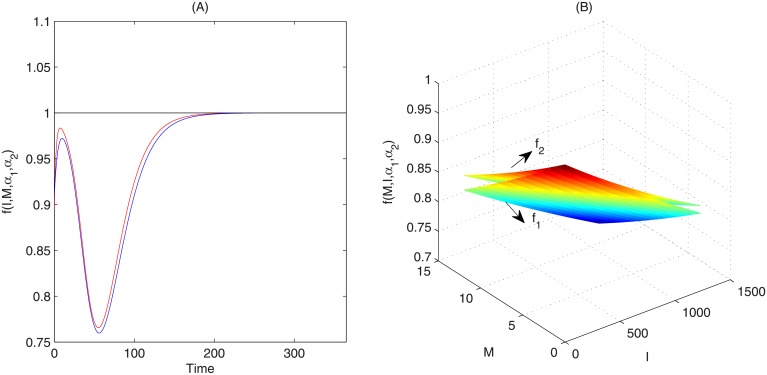
Comparison of media functions. (A) Comparison of media functions in system (1) varying with time, black curve, red curve and blue curve represent *f*_0_ = 1, *f*_1_ = *e*^−*α*_1_*I*−*α*_2_*M*^, $f_{2}=\frac{1}{1+\alpha_{1}I+\alpha_{2}M}$, respectively. (B) Comparison of *f*_1_ and *f*_2_ when parameters *α*_1_ and *α*_2_ are fixed as values estimated by using Least Square Method and *I*, *M* vary.

We plot the epidemic curves of system (1) without media function (*f*_0_ = 1) and with media function *f*_1_, *f*_2_, in Fig 3, using the parameter values listed in Table 1. This figure shows that the number of infected individuals is greatly reduced when media impact is considered. For example, the peak magnitude is reduced from 2091 to 1225 (1189) when we use the media function *f*_1_ (*f*_2_), giving a reduction of 41.4% (43.1%). The total number of infected individuals over one year is also reduced, from 87974 to 74391 (73370). It also follows from Fig 3 that there is no obvious difference on the epidemic prevalence for system (1) with media function *f*_1_ or function *f*_2_, though the number of media items looks very different for the different media functions, which may be attributed to the small numbers of media items and the small value of the weight of media effects sensitive to the media reports. This is also confirmed in Fig 4(A) and 4(B) that media functions *f*_1_ and *f*_2_ are almost the same under their estimated parameters.

**Fig 5:**
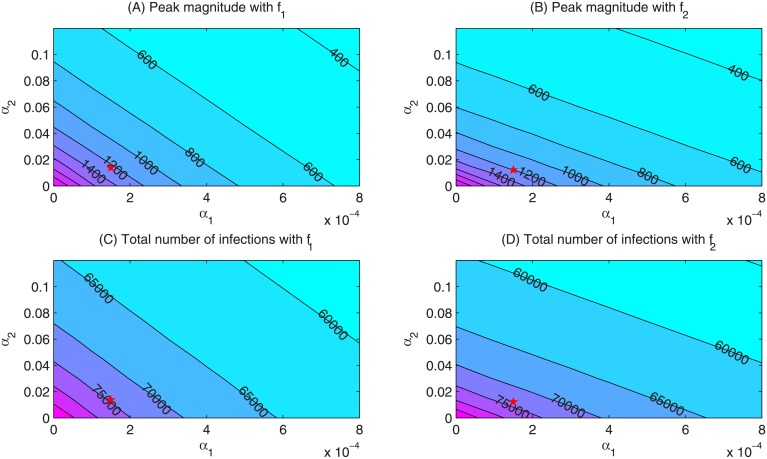
Contour plots. Contour plots of the peak magnitude and the total number of infections versus *α*_1_ and *α*_2_ by using media functions *f*_1_ and *f*_2_, respectively. The red star represents the *α*_1_ and *α*_2_ we have parameterized by using the real data.

**Fig 6:**
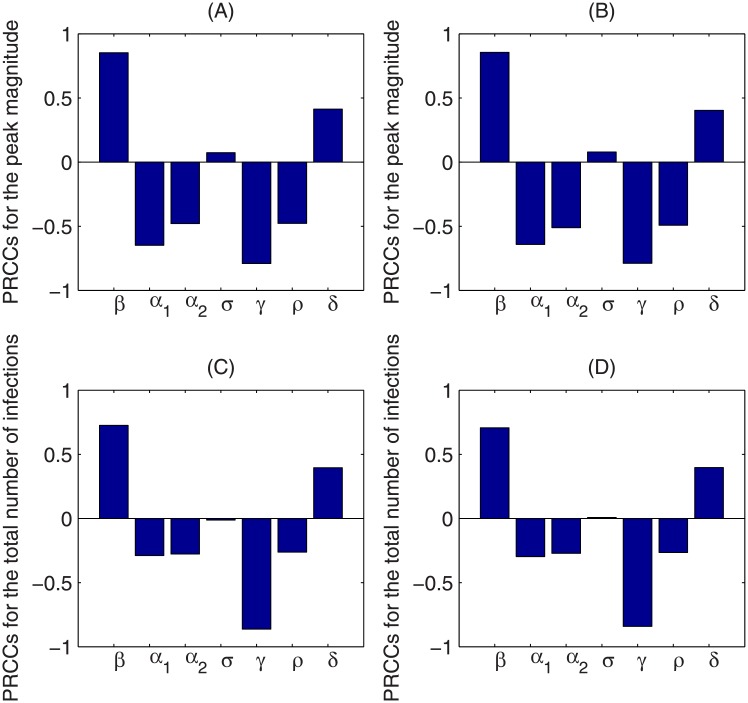
Sensitivity analysis. LHS-PRCC results for peak magnitude of infected individuals and total number of infections of system (1) with respective to *β*, *α*_1_, *α*_2_, *σ*, *γ*, *ρ*, *δ*. The sensitivity analysis is done with 2000 bins, the left and right columns correspond to system (1) with media functions *f*_1_ and *f*_2_, respectively.

**Fig 7:**
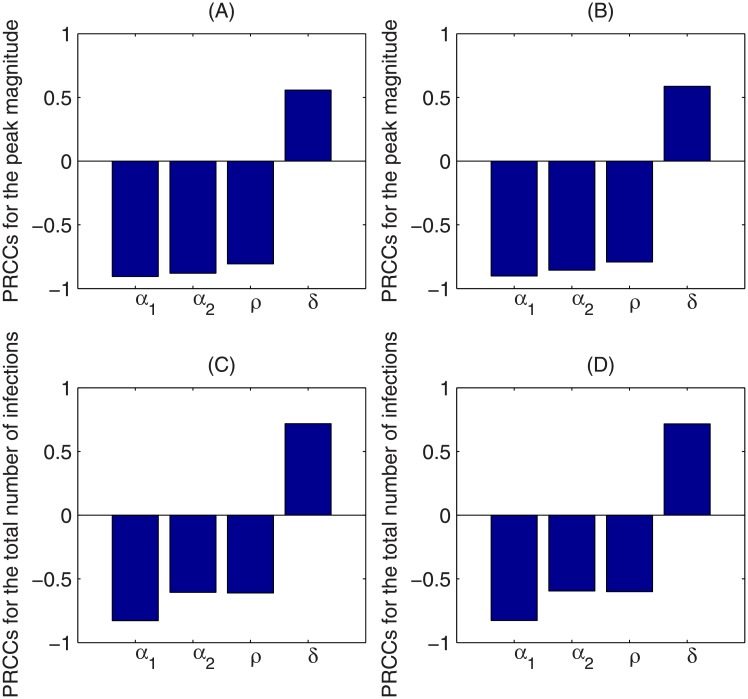
Sensitivity analysis. LHS-PRCC results for peak magnitude of infected individuals and total number of infections of system (1) with respective to *α*_1_, *α*_2_, *ρ*, *δ*. The other parameters are fixed as estimated in Table 1. The sensitivity analysis is done with 2000 bins, the left and right column correspond to system (1) with media functions *f*_1_ and *f*_2_, respectively.

The contour plots of Fig 5 show the dependence of the peak magnitude and the total number of infections on the weight of media effects sensitive to infected individuals *α*_1_ and the weight of media effects sensitive to the media reports *α*_2_, using the two different media functions. With increases in *α*_1_ or *α*_2_, both the peak magnitude of the number of infected individuals and the total number of infections over a year decrease greatly, indicating that the media effect reduces outbreak severity. To identify key parameters that influence the disease infection dynamics, we use Latin Hypercube Sampling (LHS) and partial rank correlation coefficients (PRCCs) to examine the dependence of the peak magnitude and total number of infections on corresponding model parameters. It follows from Figs 6 and 7 that, despite the fact that the baseline value of the transmission rate *β* and the recovery rate *γ* are the most sensitive parameters to the peak magnitude and the total number of infections, parameters related to the media coverage *α*_1_, *α*_2_, *ρ*, *δ* can also significantly affect the results. In particular, increases in the weight of infective cases *α*_1_ and media reports *α*_2_ in the media function will significantly reduce the peak magnitude and total number infections. Also, decreases in the media reporting rate *ρ*, and increases in the media waning rate will lead to more severe outbreaks.

**Fig 8:**
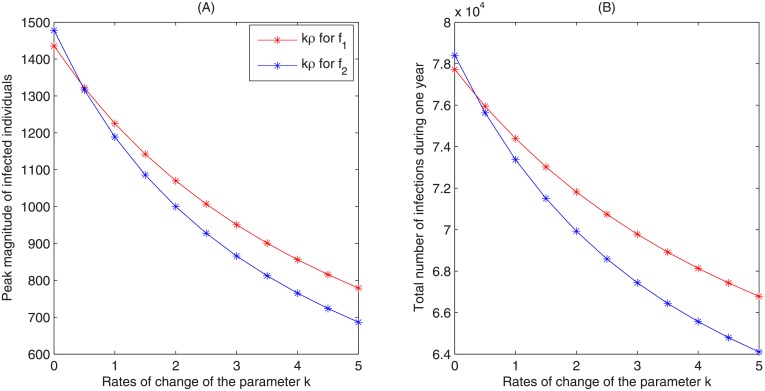
Plots of the peak magnitude and total infections by varying *k* (the rates of change of *ρ*). The red and blue curves with asterisk markers are the results by using function *f*_1_ and *f*_2_, respectively. Each column of markers denotes that *k* increases 50% per time. All the other parameters are fixed as shown in Table 1.

To access the effectiveness of enhancing the media reporting on the epidemic outbreak, we plot the variation in peak magnitude and total infections with the corresponding parameter *ρ*, as shown in Fig 8(A) and 8(B). It shows that increasing *ρ* by 4 times from baseline value (while keeping other parameters fixed) can reduce the peak magnitude from 1225 to 779 (decreased by 36.4%) for the media function *f*_1_ or 1189 to 687 (decrease by 42.2%) for the media function *f*_2_, and also can reduce the number of total infections from 74391 to 66783 for *f*_1_ or 73370 to 64102 for *f*_2_.

**Fig 9:**
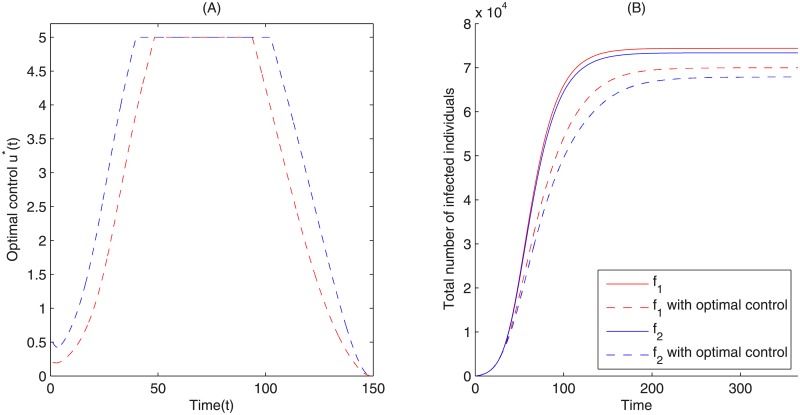
Optimal control. (A) The optimal control *u*(*t*) for the optimal control problem (4) obtained by using Forward-Backward sweep method. The red and blue dashed curves represent the optimal control for system (4) with different media functions *f*_1_ and *f*_2_, respectively. (B) Comparison of total infections for system (1) and the optimal control system (4). *A* = 1, *B* = 5, *u*_max_ = 5, *t*_0_ = 0, *t*_*end*_ = 150, all other parameters are shown in Table 1.

To further investigate what pattern of media report is optimal in minimizing the number of infected individuals and costs, we simulate the optimal control system (4) and obtain the optimal control. Here, we fix the parameter values as listed in Table 1 and employ the Forward-Backward Sweep method. It follows from Fig 9(A) that the optimal control is to continuously strengthen/increase the media reports at the beginning of an epidemic, when the disease starts to spread. A maximum level should then be maintained in times surrounding the peak number of infections, and then it should be slowly weakened as the infection reduces in the population. It is clear that the optimal control is similar for the two different media functions *f*_1_ and *f*_2_, however, the optimal media reporting intensity for system (4) with media function *f*_2_ is stronger than that for system (4) with media function *f*_1_ (i.e., $u_{2}^{*}>u_{1}^{*}$).

**Fig 10:**
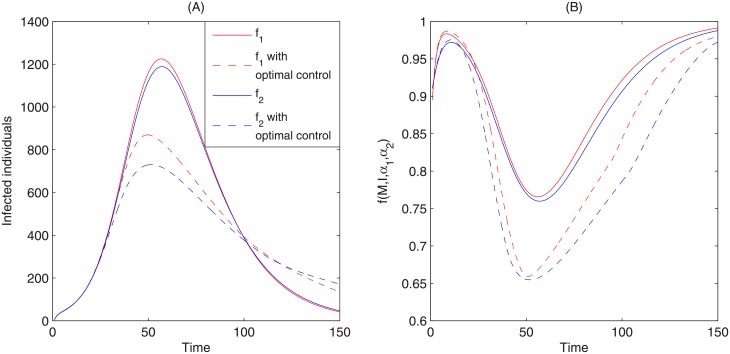
Optimal control solutions. (A) Comparison of numbers of infected individuals for system (1) and the optimal control system (4). The solid curves are solutions of the original system (1) while the dashed curves are solutions of the optimal control system (4). The red and blue colors represent the corresponding solutions by using media function *f*_1_ and *f*_2_, respectively. (B) Comparison of two different media functions *f*_1_ (red), *f*_2_ (blue) before (solid) and after (dashed) optimal control.

Figs 9(B) and 10 show the optimal epidemic curves under the optimal reporting intensity. These figures indicate that the optimal control significantly reduces the peak magnitude and total number of infected individuals. They also show that the peak magnitude appears earlier in time for both media functions *f*_1_ and *f*_2_. Comparing Fig 3 to Fig 9(B) and Fig 10(A), we see that, while simply having media reports during an epidemic can greatly reduce the severity of an epidemic, it is further mitigated under the optimal reporting intensity. Moreover, in such a scenario, a stronger media report intensity $u_{2}^{*}$ for media function *f*_2_ gives rise to a greater reduction in peak magnitude and the total number of infected individuals than the media report intensity $u_{1}^{*}$.

## Discussion

It is known that media reports can play an important role in generating public awareness and promoting disease mitigation measures. Quantifying and evaluating the media impact on the control of emerging infectious diseases is quite challenging. Our study here included the intensity of media reports as a separate compartment, and a modified transmission rate that is reduced using a media function that is bounded between 0 and 1. We also considered a novel media function which depends both on the number of infected individuals *I* and the intensity of mass media *M* (where mass media reports were assumed to depend on the reporting of newly infected individuals, *ρσE*) [8].

We calculated the basic reproduction number of system (1) which is the same as that for the classical SEIR model. This result illustrates that the mass media has no effect on the basic reproduction number, which agrees with the previous studies [11, 24, 27]. We then investigated the threshold dynamics of the proposed model with a general media function effect. We theoretically investigated the optimal control problem by seeking an optimal media reporting intensity to minimize the total infected individuals and the cost of media reporting. The optimal media reporting intensity obtained here indicated that during the early stage of epidemic we should quickly enhance the media reporting intensity, keep it at the maximum level during the time period around the peak of the epidemic, and then decrease the intensity after the epidemic vanishes significantly.

By fitting our proposed model to laboratory-confirmed case data from the 8th Hospital of Xi’an over the first 19 days of the 2009 H1N1 influenza pandemic (to ensure that the data lie in the exponential growth phase of the epidemic), we estimated the unknown model parameters and the basic reproduction numbers without media impact and with two special media functions. We found that the basic reproduction number may be underestimated if the media impact is not considered. We also illustrated that the peak magnitude of the endemic would greatly decrease when the mass media function is considered, which has also been demonstrated in [26]. Sensitivity analysis indicated that the severity of the disease outbreak is sensitive to the parameters associated with the media impact (the reporting rate *ρ*, media waning rate *δ*, weight of media effect sensitive to infected individuals *α*_1_, and weight of the media effect sensitive to media items *α*_2_) besides the epidemiological parameters (like transmission rate *β* and the recovery rate *γ*). In particular, the outbreak severity is more sensitive to the weight *α*_1_ than weight *α*_2_ (shown in Figs 5–7), indicating that more response to the number of infected individuals will lead to greater reductions in the peak magnitude and the total number of infections.

For the particular media functions *f*_1_ and *f*_2_, we observed no obvious differences in the epidemic curve (shown in Fig 3) when the optimal reporting rate was not considered, though from the point of R-square or *AIC* for the Least-Square case, the model with *f*_2_ can fit the observed data better than the model with *f*_1_. However, with the optimal media reporting rate the optimal epidemic curves are quite different. In particular, the shape of the optimal control $u_{1}^{*}$ and $u_{2}^{*}$ are similar, but $u_{2}^{*}$ is greater than $u_{1}^{*}$, causing the optimal solution of system (4) with *f*_2_ to be less than the optimal solution of system (4) with *f*_1_. This means that there is optimal media reporting function such that the number of infected individuals and costs reach the minimum, which helps design an optimal news releasing patterns that mostly affect individuals’ behaviour changes, and hence result in the infection significantly decline.

In previous studies, media campaigns have been characterized as a dynamic variable [21]. Media data can be collected to inform the mass media compartment [8, 25]. In our current study, we include a separate compartment for mass media reports. We will consider the incorporation of mass media data in future work.

In summary, we extend the classical SEIR model by incorporating the media as a separate compartment and through the modification of the transmission rate by a media factor associated with not only the number of infected individuals *I* but also the media items *M*. Through the inclusion of the media compartment and the modified transmission rate *βf*(*I*, *M*) we can use model (1) to study the effects of *I* and *M* separately. In this study, we focus our work on understanding the media impact on the transmission of 2009 H1N1 in Shaanxi, China, and explore the efficiency of optimal control on the media reporting rate. Ultimately, we find that response to the number of infected individuals *I* will lead to greater reductions in the peak magnitude and the total number of infections. We also find that the optimal media reporting intensity should be enhanced early in the outbreak, be kept at the maximum level during the time period around the peak of the epidemic, and then be decreased after the epidemic reaches a low level to ensure a minimal level of infection in a population.

## Supporting information

S1 File Stability of equilibria, calculation of the optimal control, model fits without M. (PDF)

S2 File Data from the 8th hospital of Xi’an. (XLSX)

S1 Fig Data fitting for four candidate models. (EPS)
