# Supplementary material for: Optimal media reporting intensity on mitigating spread of an emerging infectious disease
Source: PLoS One. 2019 Mar 21;14(3):e0213898. doi: 10.1371/journal.pone.0213898 (PMC6428274; doi:10.1371/journal.pone.0213898)
Supplement: S1 File — (PDF) [file pone.0213898.s001.pdf]

Supporting Information (SI): Optimal media reporting intensity on mitigating spread of an emerging infectious disease

October 31, 2018

Appendix A: Stability of equilibria

Consider the system

$$\begin{cases} \frac{dS}{dt} = \Lambda - f(I, M, \alpha_1, \alpha_2)\beta SI - \mu S, \\ \frac{dE}{dt} = f(I, M, \alpha_1, \alpha_2)\beta SI - \sigma E - \mu E, \\ \frac{dI}{dt} = \sigma E - \gamma I - \mu I, \\ \frac{dR}{dt} = \gamma I - \mu R, \\ \frac{dM}{dt} = \rho\sigma E - \delta M. \end{cases} \tag{1}$$

Define $\mathcal{D} = \{(S(t), E(t), I(t), R(t), M(t)) \in R^5_+, t \geq 0 | 0 \leq S(t) + E(t) + I(t) + R(t) \leq \frac{\Lambda}{\mu}, 0 \leq M(t) \leq \frac{\rho\sigma\Lambda}{\mu\delta}\}$. It can be shown that $\mathcal{D}$ is a positively invariant and attracting region.

Denote the total population $N(t) = S(t) + E(t) + I(t) + R(t)$, It's easy to see that $N(t)$ satisfies $\frac{dN}{dt} = \Lambda - \mu N$, from which we can have

$$N(t) = \frac{\Lambda}{\mu} + (N(0) - \frac{\Lambda}{\mu})e^{-\mu t},$$

Then $N(t) \to \frac{\Lambda}{\mu}$ as $t \to \infty$, and if $0 \leq N(0) \leq \frac{\Lambda}{\mu}$, we have $0 \leq N(t) \leq \frac{\Lambda}{\mu}$ for any $t \geq 0$. Furthermore, we have that if $S(0) > 0$, $S(t) > 0$, which can be shown by contradiction. Assume that $S(t) = 0$ for the first time at time $t_1 > 0$, then $S'(t_1) \leq 0$, which contradicts with $S'(t_1) = \Lambda > 0$ following from the first equation of system (1). Thus, we have shown that if $S(0) > 0$, then $S(t) > 0$. Similarly, it can be shown that $E(t) \geq 0$ if $E(0) \geq 0$, $I(t) \geq 0$ if $I(0) \geq 0$, $R(t) \geq 0$ if $R(0) \geq 0$ and $M(t) \geq 0$ if $M(0) \geq 0$.

Suppose $N(t) > \frac{\Lambda}{\mu}$, then $\frac{dN(t)}{dt} = \Lambda - \mu N(t) < 0$, which means that $N(t)$ approaches $\frac{\Lambda}{\mu}$ asymptotically. Now suppose $M(t) > \frac{\rho\sigma\Lambda}{\mu\delta}$, then $\frac{dM(t)}{dt} = \rho\sigma E(t) - \delta M(t) < 0$. Hence, we have that $\mathcal{D}$ is a positively invariant and attracting region in R^5_+ for system (1).

Stability of the disease free equilibrium

The Jacobian matrix of the reduced model at the disease free equilibrium $\mathbb{E}_0(\frac{\Lambda}{\mu}, 0, 0, 0)$ is

$$J(\mathbb{E}_0) = \begin{pmatrix} -\mu & 0 & -\frac{\beta\Lambda}{\mu} & 0 \\ 0 & -(\sigma+\mu) & \frac{\beta\Lambda}{\mu} & 0 \\ 0 & \sigma & -(\gamma+\mu) & 0 \\ 0 & \rho\sigma & 0 & -\delta \end{pmatrix},$$

and the corresponding characteristic eigenvalues are $-\mu, -\delta$ and the roots that satisfy the function $(\lambda + \sigma + \mu)(\lambda + \gamma + \mu) - \frac{\beta\sigma\Lambda}{\mu} = 0$. Thus, all eigenvalues have negative real part if $(\sigma + \mu)(\gamma + \mu) - \frac{\beta\sigma\Lambda}{\mu} > 0$, which is equivalent to $\mathcal{R}_0 = \frac{\beta\sigma\Lambda}{\mu(\sigma+\mu)(\gamma+\mu)} < 1$. However, positive real part eigenvalues are possible if

$(\sigma+\mu)(\gamma+\mu)-\frac{\beta\sigma\Lambda}{\mu}<0$, being equivalent to $\mathcal{R}_0>1$. Hence the disease free equilibrium $\mathbb{E}_0$ is locally asymptotically stable if $\mathcal{R}_0<1$, and unstable if $\mathcal{R}_0>1$.

Let $V=E+\frac{\sigma+\mu}{\sigma}I$, then if $\mathcal{R}_0\leq 1$, the derivative of V along a solution of (1) is

$$\begin{aligned} V'=E'+\tfrac{\sigma+\mu}{\sigma}I' &= f(I,M)\beta SI-\tfrac{(\gamma+\mu)(\sigma+\mu)}{\sigma}I \\ &= \tfrac{(\gamma+\mu)(\sigma+\mu)}{\sigma}I[\tfrac{\beta\sigma S}{(\gamma+\mu)(\sigma+\mu)}f(I,M)-1] \\ &\leq \tfrac{(\gamma+\mu)(\sigma+\mu)}{\sigma}I(\mathcal{R}_0-1)\leq 0 \end{aligned} \tag{2}$$

since $S\leq\frac{\Lambda}{\mu}, I\geq 0$ and $0\leq f(I,M)\leq 1$. $V'=0$ holds true only when $I=0$. Thus we have that the disease free equilibrium $\mathbb{E}_0$ is globally asymptotically stable when $\mathcal{R}_0\leq 1$.

Stability of the endemic equilibrium

When $\mathcal{R}_0>1$, the unique endemic equilibrium $\mathbb{E}^*$ exists and the disease free equilibrium $\mathbb{E}_0$ is unstable. The Jacobian matrix of the reduced model at $\mathbb{E}^*$ is

$$J(\mathbb{E}^*)=\begin{pmatrix} -\beta I^*f(I^*,M^*)-\mu & 0 & -\beta S^*f(I^*,M^*)-\beta S^*I^*\frac{\partial f(I^*,M^*)}{\partial I} & -\beta S^*I^*\frac{\partial f(I^*,M^*)}{\partial M} \\ \beta I^*f(I^*,M^*) & -(\sigma+\mu) & \beta S^*f(I^*,M^*)+\beta S^*I^*\frac{\partial f(I^*,M^*)}{\partial I} & \beta S^*I^*\frac{\partial f(I^*,M^*)}{\partial M} \\ 0 & \sigma & -(\gamma+\mu) & 0 \\ 0 & \rho\sigma & 0 & -\delta \end{pmatrix},$$

and the corresponding characteristic equation is

$$\lambda^4+a_1\lambda^3+a_2\lambda^2+a_3\lambda+a_4=0,$$

where

$$\begin{aligned} a_1 &= \beta I^*f(I^*,M^*)+\mu+\delta+\sigma+\mu+\gamma+\mu>0, \\ a_2 &= (\beta I^*f(I^*,M^*)+\mu)(\delta+\sigma+\mu+\gamma+\mu)+\delta(\sigma+\mu+\gamma+\mu)-\sigma\beta S^*I^*\tfrac{\partial f(I^*,M^*)}{\partial I}-\rho\sigma\beta S^*I^*\tfrac{\partial f(I^*,M^*)}{\partial M}>0, \\ a_3 &= (\beta I^*f(I^*,M^*)+\mu)\delta(\sigma+\mu+\gamma+\mu)+\beta I^*f(I^*,M^*)(\sigma+\mu)(\gamma+\mu) \\ &\quad -(\delta+\mu)\sigma\beta S^*I^*\tfrac{\partial f(I^*,M^*)}{\partial I}-(\gamma+2\mu)\rho\sigma\beta S^*I^*\tfrac{\partial f(I^*,M^*)}{\partial M} \\ &> 0, \\ a_4 &= \beta I^*f(I^*,M^*)\delta(\sigma+\mu)(\gamma+\mu)-\mu\delta\sigma\beta S^*I^*\tfrac{\partial f(I^*,M^*)}{\partial I}-\mu(\gamma+\mu)\rho\sigma\beta S^*I^*\tfrac{\partial f(I^*,M^*)}{\partial M}>0. \end{aligned}$$

from which we have $a_1a_2-a_3>0$, and $a_3(a_1a_2-a_3)-a_1^2a_4>0$. Thus, by the Hurwitz stability criterion, we know that the real parts of the eigenvalues of $J(\mathbb{E}^*)$ are negative, indicating the local stability of the endemic equilibrium $\mathbb{E}^*$.

When we prove the local stability of the endemic state we have the following detailed calculation.

$$\begin{aligned} a_1a_2-a_3 &= (\beta I^*f(I^*,M^*)+\mu)^2(\delta+\sigma+\mu+\gamma+\mu)+(\beta I^*f(I^*,M^*)+\mu)(\delta+\sigma+\mu+\gamma+\mu)^2 \\ &\quad +\delta(\sigma+\mu+\gamma+\mu)(\delta+\sigma+\mu+\gamma+\mu)-\beta I^*f(I^*,M^*)(\sigma+\mu)(\gamma+\mu) \\ &\quad -(\beta I^*f(I^*,M^*)+\mu+\sigma+\mu+\gamma)\sigma\beta S^*I^*\tfrac{\partial f(I^*,M^*)}{\partial I}-(\beta I^*f(I^*,M^*)+\mu+\delta+\sigma)\rho\sigma\beta S^*I^*\tfrac{\partial f(I^*,M^*)}{\partial M} \\ &> 0, \end{aligned}$$

and

$$\begin{aligned} a_3(a_1a_2-a_3)-a_1^2a_4 &= A_1(\beta I^*f(I^*,M^*))^3+A_2(\beta I^*f(I^*,M^*))^2+A_3\beta I^*f(I^*,M^*)+A_4 \\ &\quad +B\sigma\beta S^*I^*\tfrac{\partial f(I^*,M^*)}{\partial I}+C\rho\sigma\beta S^*I^*\tfrac{\partial f(I^*,M^*)}{\partial M}+D \\ &> 0, \end{aligned}$$

where 31

$$
\begin{aligned}
A_1 &= \delta(\sigma+\mu+\gamma+\mu)(\delta+\sigma+\mu+\gamma+\mu)+(\sigma+\mu)(\gamma+\mu)(\sigma+\mu+\gamma+\mu)>0,\\
A_2 &= 3\mu\delta(\sigma+\mu+\gamma+\mu)(\delta+\sigma+\mu+\gamma+\mu)+2\mu(\sigma+\mu)(\gamma+\mu)(\sigma+\mu+\gamma+\mu)\\
&\quad +\delta(\sigma+\mu+\gamma+\mu)(\delta+\sigma+\mu+\gamma+\mu)^2+(\sigma+\mu)^3(\gamma+\mu)+(\sigma+\mu)(\gamma+\mu)^3\\
&\quad +(\sigma+\mu)^2(\gamma+\mu)^2-\delta(\sigma+\mu)(\gamma+\mu)(\delta+\sigma+\mu+\gamma+\mu)\\
&> 0,\\
A_3 &= 3\mu^2\delta(\sigma+\mu+\gamma+\mu)(\delta+\sigma+\mu+\gamma+\mu)+\mu^2(\sigma+\mu)(\gamma+\mu)(\sigma+\mu+\gamma+\mu)\\
&\quad +2\mu\delta(\sigma+\mu+\gamma+\mu)(\delta+\sigma+\mu+\gamma+\mu)^2+\mu(\sigma+\mu)(\gamma+\mu)(\delta+\sigma+\mu+\gamma+\mu)^2\\
&\quad +\delta^2(\sigma+\mu+\gamma+\mu)^2(\delta+\sigma+\mu+\gamma+\mu)-\mu\delta(\sigma+\mu)(\gamma+\mu)(\sigma+\mu+\gamma+\mu)\\
&\quad -\delta(\sigma+\mu)(\gamma+\mu)(\delta+2\mu)\\
&> 0,\\
A_4 &= \mu^3\delta(\sigma+\mu+\gamma+\mu)(\delta+\sigma+\mu+\gamma+\mu)+\mu^2\delta(\sigma+\mu+\gamma+\mu)(\delta+\sigma+\mu+\gamma+\mu)^2\\
&\quad +\mu\delta^2(\sigma+\mu+\gamma+\mu)^2(\delta+\sigma+\mu+\gamma+\mu)\\
&> 0,\\
B &= -[(\delta+\sigma+\mu+\gamma+\mu)(\mu+\delta)+\delta(\sigma+\mu+\gamma)+(\sigma+\mu)(\gamma+\mu)](\beta I^* f(I^*,M^*))^2\\
&\quad -[2\mu^2(\delta+\sigma+\mu+\gamma+\mu)+(\mu+\delta)(\delta+\mu+\sigma+\gamma+\mu)^2+2\mu\delta(\sigma+\mu+\gamma)+\delta(\sigma+\mu)^2\\
&\quad +\delta\gamma(\sigma+\mu+\gamma+\mu)+(\sigma+\mu)(\gamma+\mu)(\sigma+\mu+\gamma)]\beta I^* f(I^*,M^*)\\
&\quad -[\mu^2(2\mu+\sigma+\gamma+\mu)(\delta+\mu+\gamma+\sigma+\mu)+\delta(\mu+\delta)(\sigma+\mu+\gamma+\mu)(\delta+\mu+\sigma+\mu+\gamma)\\
&\quad +\mu\delta(\sigma+\mu+\gamma)(2\mu+\sigma+\gamma+\mu)]\\
&< 0,\\
C &= -[(2\mu+\gamma)(\delta+\sigma+\mu+\gamma+\mu)+\delta(\sigma+\mu+\gamma+\mu)+\sigma(\gamma+\mu)]f(I^*,M^*))^2\\
&\quad -[2\mu^2(\delta+\sigma+\mu)+\mu(\delta+\sigma+\mu+\gamma+\mu)^2+(\gamma+\mu)(\delta+\sigma+\mu)(\delta+\mu+\sigma+\gamma+\mu)\\
&\quad +(\mu+\gamma)^2(\delta+\gamma+\mu)+(\sigma+\mu)(\gamma+\mu)(\delta+\sigma)+\delta(\delta+\mu+\sigma+\mu)(\sigma+\mu+\gamma+\mu)]\beta I^* f(I^*,M^*)\\
&\quad -[\mu^3(\delta+\sigma+\mu)+\mu^2(\delta+\sigma+\mu)(\delta+\sigma+\mu+\gamma+\mu)+\mu\delta(\delta+\sigma+\mu)(\sigma+\mu+\gamma+\mu)\\
&\quad +\delta(2\mu+\gamma)(\sigma+\mu+\gamma+\mu)(\delta+\sigma+\mu+\gamma+\mu)]\\
&< 0,\\
D &= [(\mu+\beta I^* f(I^*,M^*)+\sigma+\mu+\gamma)\sigma\beta S^* I^* \tfrac{\partial f(I^*,M^*)}{\partial I}+(\mu+\beta I^* f(I^*,M^*)+\sigma+\delta)\rho\sigma\beta S^* I^* \tfrac{\partial f(I^*,M^*)}{\partial M}]\\
&\quad *[(\mu+\delta)\sigma\beta S^* I^* \tfrac{\partial f(I^*,M^*)}{\partial I}+(\mu+\gamma+\mu)\rho\sigma\beta S^* I^* \tfrac{\partial f(I^*,M^*)}{\partial M}]\\
&> 0.
\end{aligned}
$$

Appendix B: Calculation of the optimal control 32

Consider the optimal control problem to minimize the objective functional 33

$$J(u)=\int_{t_0}^{T}[AI(t)+\tfrac{B}{2}u^2(t)]dt \tag{3}$$

subject to 34

$$
\begin{cases}
\frac{dS}{dt}=\Lambda-\beta f(I,M)SI-\mu S, \quad S(t_0)=S_0>0,\\
\frac{dE}{dt}=\beta f(I,M)SI-\sigma E-\mu E, \quad E(t_0)=E_0\geq 0,\\
\frac{dI}{dt}=\sigma E-\gamma I-\mu I, \quad I(t_0)=I_0\geq 0,\\
\frac{dR}{dt}=\gamma I-\mu R, \quad R(t_0)=R_0\geq 0,\\
\frac{dM}{dt}=u(t)\rho\sigma E-\delta M, \quad M(t_0)=M_0\geq 0.
\end{cases} \tag{4}
$$

Define the Hamiltonian H for the control problem: 35

$$H(S,I,R,M,u,\lambda_S,\lambda_I,\lambda_R,\lambda_M,t)=AI(t)+\tfrac{B}{2}u^2(t)+\lambda_S\tfrac{dS(t)}{dt}+\lambda_E\tfrac{dE(t)}{dt}+\lambda_I\tfrac{dI(t)}{dt}+\lambda_R\tfrac{dR(t)}{dt}+\lambda_M\tfrac{dM(t)}{dt}. \tag{5}$$

then the adjoint equations and transversality conditions are given:

$$\begin{cases} \lambda'_S(t) = -\frac{\partial H}{\partial S} = \beta f(I,M)I(\lambda_S - \lambda_E) + \mu\lambda_E, & \lambda_S(t_{end}) = 0, \\ \lambda'_E(t) = -\frac{\partial H}{\partial E} = \sigma(\lambda_E - \lambda_I - \rho u(t)\lambda_M) + \mu\lambda_E, & \lambda_E(t_{end}) = 0, \\ \lambda'_I(t) = -\frac{\partial H}{\partial I} = -A + \beta f(I,M)S(\lambda_S - \lambda_E) + \beta SI\frac{\partial f(I,M)}{\partial I}(\lambda_S - \lambda_E) + \gamma(\lambda_I - \lambda_R) + \mu\lambda_I, & \lambda_I(t_{end}) = 0, \\ \lambda'_R(t) = -\frac{\partial H}{\partial R} = \mu\lambda_R, & \lambda_R(t_{end}) = 0, \\ \lambda'_M(t) = -\frac{\partial H}{\partial M} = \beta SI\frac{\partial f(I,M)}{\partial M}(\lambda_S - \lambda_E) + \delta\lambda_M, & \lambda_M(t_{end}) = 0. \end{cases} \tag{6}$$

By the optimality conditions, we have

$$\frac{\partial H}{\partial u} = Bu + \lambda_M\rho\sigma E = 0 \quad \Rightarrow u^* = -\frac{\lambda_M\rho\sigma E}{B}. \tag{7}$$

Note that the boundness are placed on the control variable $u(t)$, $0 \leq u(t) \leq u_{max}$, then the optimality condition is changed to

$$u^*(t) = \begin{cases} 0, & \text{if } \frac{\partial H}{\partial u} > 0, \text{i.e. } \frac{-\lambda_M\rho\sigma E}{B} < 0, \\ \frac{-\lambda_M\rho\sigma E}{B}, & \text{if } \frac{\partial H}{\partial u} = 0, \text{i.e. } 0 \leq \frac{-\lambda_M\rho\sigma E}{B} \leq u_{max}, \\ C, & \text{if } \frac{\partial H}{\partial u} < 0, \text{i.e. } \frac{-\lambda_M\rho\sigma E}{B} > u_{max}. \end{cases} \tag{8}$$

This can be rewritten in compact notation

$$u^*(t) = \max\{\min\{\frac{-\lambda_M\rho\sigma E}{B}, u_{max}\}, 0\}.$$

which is the optimal control.

Appendix C: Model selection

In our paper, considering the fact that the media campaigns can be characterized as a dynamic variable, we propose a model incorporating the media reports as a separate compartment and the transmission rate is modified by a media function affected by the media reports and the number of infected individuals. For completeness, we propose 4 candidate infectious models and use AIC based on Least Square Method for model selection. The four models are shown in the following:

Model 1:

$$\begin{cases} \frac{dS}{dt} = -\beta e^{-\alpha_1 I}SI, \\ \frac{dE}{dt} = \beta e^{-\alpha_1 I}SI - \sigma E, \\ \frac{dI}{dt} = \sigma E - \gamma I, \\ \frac{dR}{dt} = \gamma I. \end{cases} \tag{9}$$

Model 2:

$$\begin{cases} \frac{dS}{dt} = -\beta\frac{1}{1+\alpha_1 I}SI, \\ \frac{dE}{dt} = \beta\frac{1}{1+\alpha_1 I}SI - \sigma E, \\ \frac{dI}{dt} = \sigma E - \gamma I, \\ \frac{dR}{dt} = \gamma I. \end{cases} \tag{10}$$

Model 3:

$$\begin{cases} \frac{dS}{dt} = -\beta e^{-\alpha_1 I - \alpha_2 M}SI, \\ \frac{dE}{dt} = \beta e^{-\alpha_1 I - \alpha_2 M}SI - \sigma E, \\ \frac{dI}{dt} = \sigma E - \gamma I, \\ \frac{dR}{dt} = \gamma I, \\ \frac{dM}{dt} = \rho\sigma E - \delta M. \end{cases} \tag{11}$$

Model 4:

$$\begin{cases} \frac{dS}{dt} = -\beta\frac{1}{1+\alpha_1 I+\alpha_2 M}SI, \\ \frac{dE}{dt} = \beta\frac{1}{1+\alpha_1 I+\alpha_2 M}SI - \sigma E, \\ \frac{dI}{dt} = \sigma E - \gamma I, \\ \frac{dR}{dt} = \gamma I, \\ \frac{dM}{dt} = \rho\sigma E - \delta M. \end{cases} \tag{12}$$

In the first two models, the transmission rate is modified by a media function only related to the number of infected individuals. In the latter two models, which are the two particular models proposed in our paper, the dynamics of media reports is considered and the media function is a decreasing function with respect to the number of infected individuals (I) and the media reports (M). We use the data from 3rd September to 21st September and the Least Square method to estimate the parameter values and AIC values, the data fitting for four different models are shown in the following Figure S1 and Table S1.

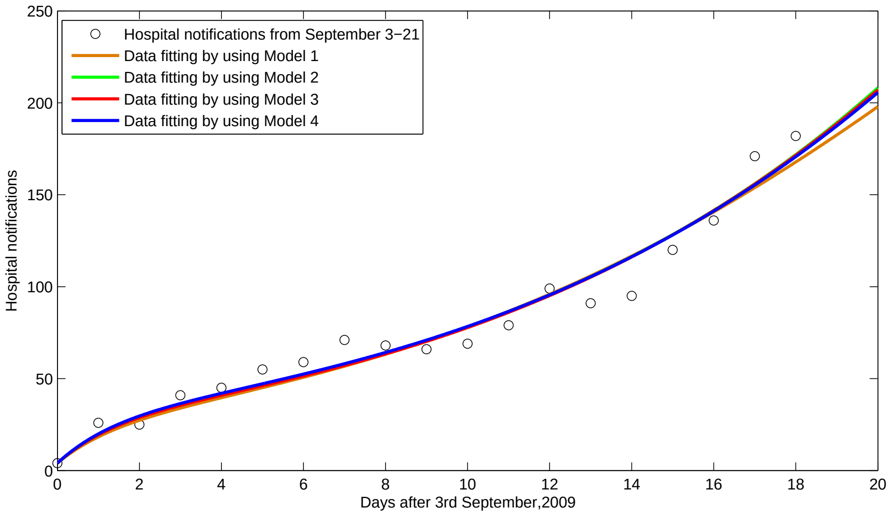

S1 Fig. Data fitting for four models. Circles represent the hospital notifications from 3rd September to 21th September in the 8th hospital of Xi'an, orange, green, red, blue curves are the fitting curves for the Model 1, Model 2, Model 3, and Model 4, respectively.

S1 Table. R-square and AIC values for four candidate models.

	Model 1	Model 2	Model 3	Model 4
R-square	0.9506	0.9514	0.9577	0.9583
AIC	96.3229	96.0260	99.3676	99.0981

We see that the model without the M compartment has a slightly better fit. However, as we are interested in study the effects of M and I in later stages of the outbreak, we have elected to include an in-depth study of the model with M compartment and media functions f_1 and f_2.
